# Physiological and Biochemical Responses of *Solanum lycopersicum* L. to Benzo[a]pyrene Contaminated Soils

**DOI:** 10.3390/ijms24043741

**Published:** 2023-02-13

**Authors:** Marina Voloshina, Vishnu D. Rajput, Natalia Chernikova, Tatiana Minkina, Evgeniy Vechkanov, Saglara Mandzhieva, Mark Voloshin, Maria Krepakova, Tamara Dudnikova, Svetlana Sushkova, Andrey Plotnikov

**Affiliations:** 1 Academy of Biology and Biotechnology, Southern Federal University, Rostov-on-Don 344090, Russia; 2 Moscow Clinical Scientific Center Named after Loginov MHD, Moscow 111123, Russia

**Keywords:** antioxidant enzymes, metabolic changes, reactive oxygen species, stress marker, structural changes in tissues

## Abstract

Benzo[a]pyrene (BaP) is noted as one of the main cancer-causing pollutants in human beings and may damage the development of crop plants. The present work was designed to explore more insights into the toxic effects of BaP on *Solanum lycopersicum* L. at various doses (20, 40, and 60 MPC) spiked in Haplic Chernozem. A dose-dependent response in phytotoxicity were noted, especially in the biomass of the roots and shoots, at doses of 40 and 60 MPC BaP and the accumulation of BaP in *S. lycopersicum* tissues. Physiological and biochemical response indices were severely damaged based on applied doses of BaP. During the histochemical analysis of the localization of superoxide in the leaves of *S. lycopersicum*, formazan spots were detected in the area near the leaf’s veins. The results of a significant increase in malondialdehyde (MDA) from 2.7 to 5.1 times, proline 1.12- to 2.62-folds, however, a decrease in catalase (CAT) activity was recorded by 1.8 to 1.1 times. The activity of superoxide dismutase (SOD) increased from 1.4 to 2, peroxidase (PRX) from 2.3 to 5.25, ascorbate peroxidase (APOX) by 5.8 to 11.5, glutathione peroxidase (GP) from 3.8 to 7 times, respectively. The structure of the tissues of the roots and leaves of *S. lycopersicum* in the variants with BaP changed depending on the dose: it increased the intercellular space, cortical layer, and the epidermis, and the structure of the leaf tissues became looser.

## 1. Introduction

The soil due to its physicochemical properties can accumulate pollutants and be a source of contamination of agricultural products [1]. One of the most common classes of pollutants are polycyclic aromatic hydrocarbons (PAHs), which are formed as a result of the pyrolysis of minerals in an oxygen-limited environment or the incomplete combustion of organic matter containing carbon and hydrogen [2]. The most carcinogenic is benzo[a]pyrene (BaP), which is assigned to the first hazard class, and its content in food is unacceptable and is subjected to control in the ecosphere [3].

The accumulation of BaP in all environmental objects including soils and plants is due to anthropogenic load. The mobility of BaP in the soil and the accumulation in plant tissues depend mainly on its solubility as well as on the physiological state of plants. The background content of BaP in most mineral soils ranges from 0.1 to 5 µg/kg, while some peat and Chernozem soils are categorized by a higher level of BaP of 15–20 µg/kg [4].

BaP is lipophilic, degraded by organic solvents, and easily penetrates cell membranes [5]. It is highly resistant to biodegradation, due to which it biomagnifies quickly in the food chain by accumulating in the intercellular fluid of plants. It has been shown that BaP and 1,2-benzacenaphthene can be included in the metabolic chains of plants by binding to glucose and glutathione (GSH), penetrating into the space of the cell wall or cell organelles, causing ultrastructural damage and provoking an increase in the intensity of oxidative stress [6,7].

To study the mechanism of action of PAHs, in particular BaP, on the physiological and biochemical parameters of plants [8], it is necessary to study the work of antioxidant enzymes, photosynthetic pigments, and changes at the cellular level. At the moment, only a few aspects of the impact of PAHs on the physio-biochemical indices of plants have been explored [4]. Most of them are devoted to the toxic effects of petroleum products and other PAHs [4,9,10]. The toxic effect of BaP on the physiological and biochemical parameters is to disrupt the cellular structure, reduce total chlorophyll, and activate peroxidases and the glutathione cascade.

In recent years, research on vegetables has increased worldwide due to their nutritional value. Tomato (*Solanum lycopersicum* L.) production and consumption are constantly increasing [11]. *S. lycopersicum* is an economically important vegetable plant and ranks seventh in the worldwide production of agricultural crops [12]. Furthermore, this plant is a model species for introducing agronomically important genes into dicotyledonous crop plants [13]. The Rostov region ranks second in Russia in terms of cultivated areas of agricultural crops, one of which is *S. lycopersicum*. Additionally, tomato is one of the most cultivated crops in close proximity to the Novocherkassk Hydroelectric Power Station, the main source of PAH emissions in the Rostov region, Russia. *S. lycopersicum* is highly sensitive to the toxic effects of PAHs, in particular BaP, and is able to accumulate them.

The comprehensive assessment of plant AS performance plays a key role in monitoring, the risk assessment for crops, and the development of remediation strategies in the future. However, comprehensive studies of the physiological and biochemical state of extensive horticultural crops are not enough to provide exhaustive conclusions about the nature of the effect of BaP on *S. lycopersicum*. This work is devoted to the study of the effect of different doses of BaP on the intensity of free radical processes and the state of the enzymatic AS of *S. lycopersicum*.

## 2. Results

### 2.1. Uptake of BaP in Plant Tissues and Impact on Morphometric Parameters

**Figure 1:**
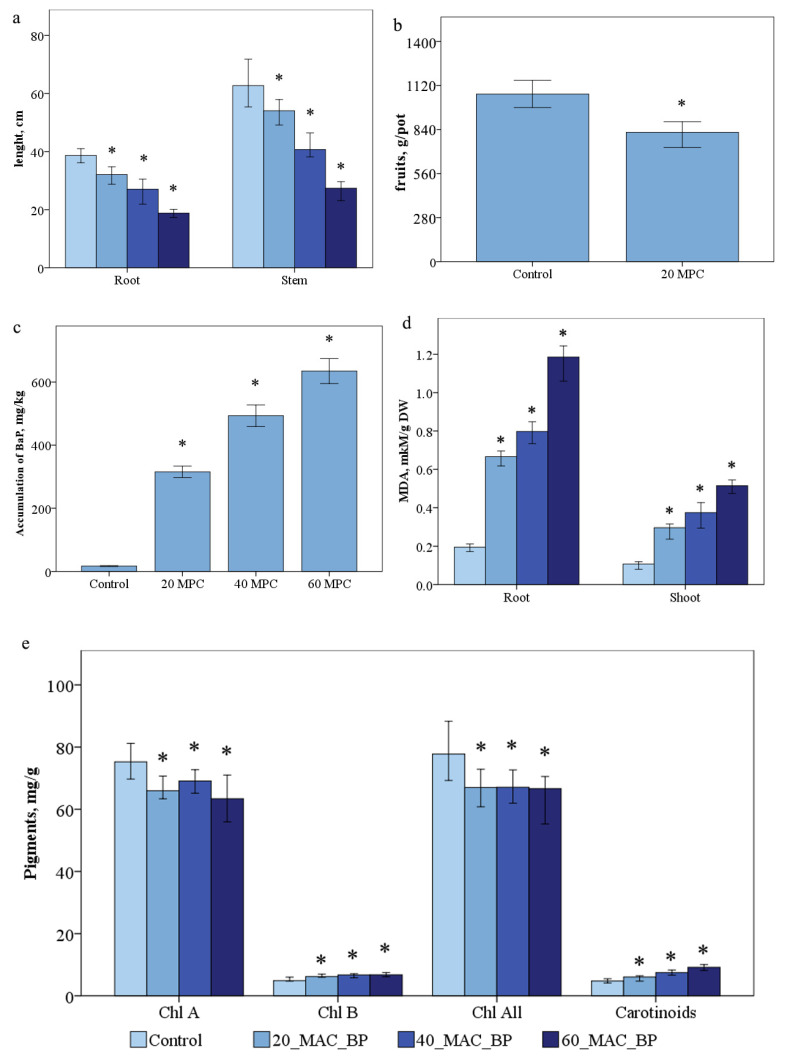
Effect of BaP on (**a**) accumulation, (**b**,**c**) morphobiometric parameters of plants, (**d**) MDA, (**e**) pigments in *Solanum lycopersicum* plants. Statistically significant differences (*p* ≤ 0.05) comparing the treated plants to the control plants are marked with an asterix (*).

As a result of measuring the morphobiometric parameters of *S. lycopersicum*, a direct pattern of inhibition of plant growth and development depending on the applied doses of the pollutant was revealed. The average length of the root and shoot was 39.1 cm and 61.4 cm, respectively, compared with the control variant. The inhibition of root growth in the variant of 20 MPC BaP was 1.2 times, and that of the shoots was 1.1 times; at 40 MPC BaP, the length of the root decreased by 1.45, and that of the shoots by 1.4 (Figure 1a). There was a decrease in tomato weight by 1.3 times in the variant of the experiment 20 MPC BaP, and in the variants of the experiment 40 and 60 MPC BaP, there was no fruiting phase (Figure 1b).

The accumulation of BaP in the control samples was 17.0 ng/g. In the variants with pollution, the increase in the accumulation of BaP in plants occurred with an increase in the dose of applying the pollutant to the soil: in the variant of the experiment 20 MPC BaP—18 times, 40 MPC BaP—30 times, and 60 MPC BaP—35 times (Figure 1c).

### 2.2. Assessment of Photosynthetic Pigments

**Figure 2:**
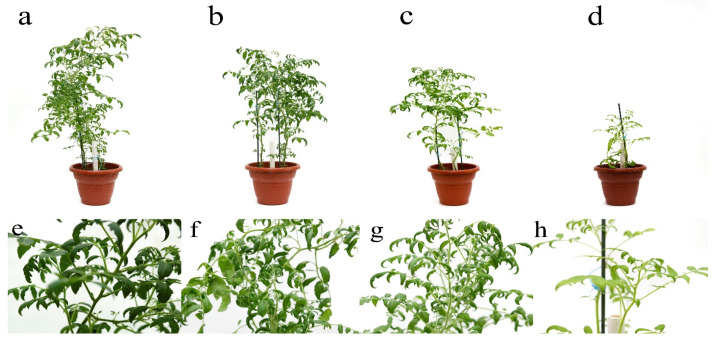
Effect of BaP on the growth, development, and deformation of the *Solanum lycopersicum* leaf blades in the active growth phase: (**a**,**e**), control; (**b**,**f**) 20 MPC BaP; (**c**,**g**) 40 MPC BaP; (**d**,**h**) 60 MPC BaP.

The content of carotene, chlorophyll a, chlorophyll b, and total chlorophyll content in the control plants were 4.9 ± 0.2, 74.2 ± 3.5, 4.8 ± 0.2, and 78.4 ± 3.4 mg/kg (Figure 1e), respectively. Thus, there was a decrease in the amount of chlorophyll *a* by 10.7%, 15%, and 21.8% and total chlorophyll by 13.4% 15%, and 19% in the experimental variants 20, 40, and 60 MPC BaP, respectively. However, chlorophyll b increased by 27%, 37%, and 42%; carotenoids by 16%, 50% and 88% in the variants 20 MPC BaP, 40 MPC BaP, and 60 MPC BaP, respectively (Figure 2e-h).

### 2.3. Assessment of Localization of Superoxide in Situ

**Figure 3:**
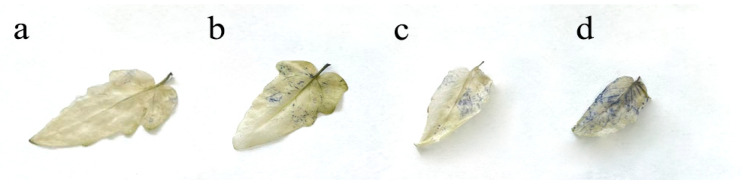
Histochemical determination of superoxide in leaves: (**a**) control; (**b**) 20 MPC BaP; (**c**) 40 MPC BaP; (**d**) 60 MPC BaP.

**Figure 4:**
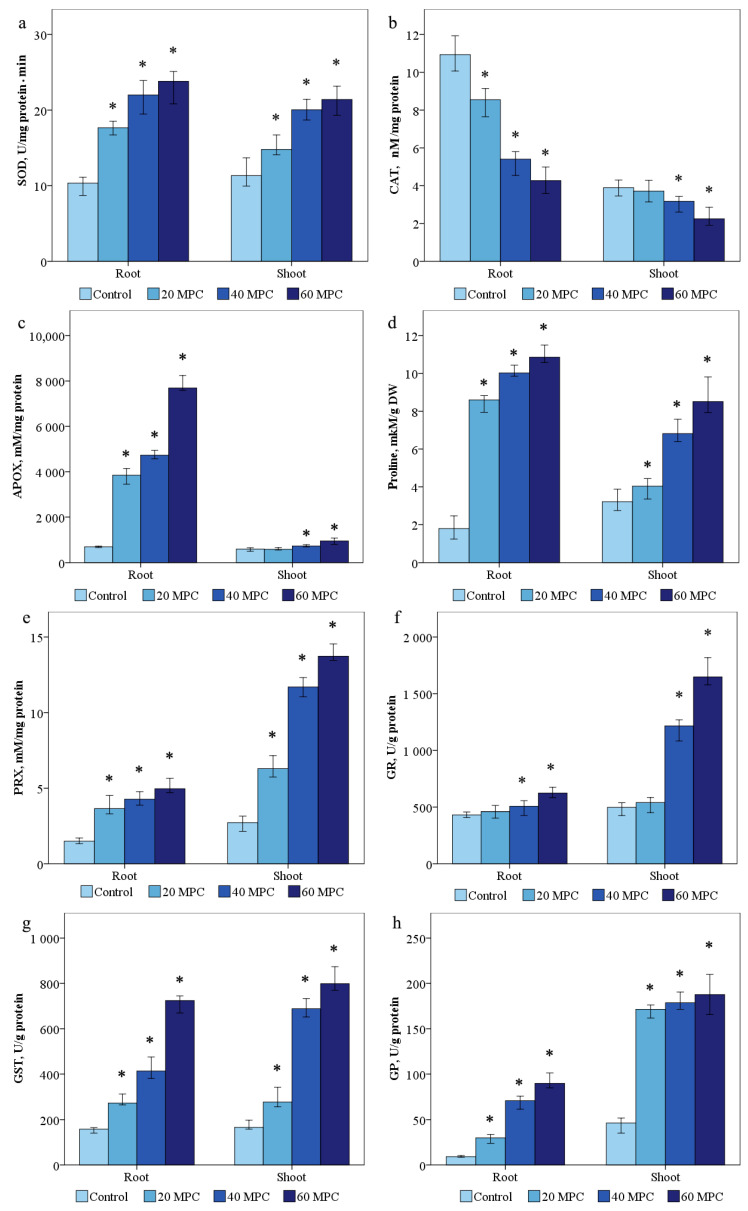
Effect of BaP on (**a**) SOD, (**b**) catalase, (**c**) proline, (**d**) PRX; (**e**) APOX, (**f**) GR, (**g**) GST, (**h**) GP in *Solanum lycopersicum* plants. Statistically significant differences (*p* ≤ 0.05) comparing the treated plants to the control plants are marked with an asterix (*).

The production of O_2_^•–^ in the leaf blade of *S. lycopersicum* was determined on the basis of the formation of blue spots (Figure 3) resulting from the reaction of NBT with O_2_^•–^. This regularity in the accumulation of superoxide was also confirmed by an increase in the amount of SOD in plants (Figure 4a).

### 2.4. Enzymatic Activity in Plants of Model Experience

The dependence of the MDA content in the leaves and roots on the concentration of the introduced pollutant was established (Figure 1d). The mean content of MDA in the shoots of plants with the control treatment was 0.11 ± 0.003 and 0.19 ± 0.002 mM/g dry weight, respectively. The largest amount of MDA was recorded in the variant of the experiment 60 MPC BaP as 4.3 times. The concentration of MDA in the shoots was increased by 2.7 times in the variants of BaP 20 MAC, 3.5 times in BaP 40 MAC, and 5.1 times in BaP 60 MAC with respect to the control (Figure 1d). In the roots, a dose-dependent increase in MDA by 3.5, 4.2, and 6.2 times was also observed at 20, 40, and 60 MPC BaP, respectively (Figure 1d).

SOD activity was 10.7 ± 0.9 and 9.9 ± 0.8 U/min·mg protein was determined in the control samples. It was shown that the activity of SOD in the shoots of the studied samples increased by 1.4 times in the variant of 20 MPC BaP, 1.8—MPC BaP times, and 60 MPC BaP—2 times. In the roots, a similar trend was observed of 1.9, 2.3, and 2.4 in the variants of 20, 40, and 60 MPC BaP, respectively (Figure 4a). 

In the control, the content of CAT averaged 4 ± 0.4 nM/mg protein in the shoots and 10.8 ± 0.7 nM/mg protein in the roots (Figure 4b). The activity of CAT in the aerial part decreased by 1.1, 1.3, and 1.8 times in the groups of 20, 40, and 60 MPC BaP, respectively. A similar picture was observed in the activity of CAT in the roots with a decrease of 1.3, 1.95, and 2.7 times in all groups.

The activity of ascorbate peroxidase in the control plant sample was 552.2 ± 9.6 and 663.6 ± 20.2 mM/mg protein in the shoots and roots was noted, respectively (Figure 4c). A significant increase in the activity of the enzyme in the roots by 5.8, 6.9, and 11.5 times was obtained in the experimental variants of 20, 40, and 60 MPC BaP, respectively. In the shoots, the difference was not so significant by 1.7, 2.1, and 3.9 times in the variants of 20, 40, and 60 MPC BaP, respectively.

The content of proline in the control *S. lycopersicum* was 3.3 ± 0.09 mM/g DW in the shoots and 2.1 ± 0.07 mM/g DW in the roots (Figure 4d). Proline in the shoots increased by 1.2, 2.1, and 2.6 times in all groups, and in the roots in variants of 20, 40, and 60 MPC BaP, by 4, 4.7, and 5.6 times, than in the control group, respectively (Figure 4d).

The average content of PRX in the shoots and roots was 2.6 ± 0.1 and 1.4 ± 0.06 (Figure 4e) f.o.p/mg, respectively, in the control variant of the experiment. In the experimental variants 20 MPC BaP, 40 MPC BaP, and 60 MPC pub, the activity of PRX increased 2.3, 3.9, and 5.25 times in the shoots, and 2.5, 2.7, and 3 times in the roots, respectively.

The activity of glutathione reductase was higher in the roots and shoots due to the presence of 20 MPC BaP, 40 MPC BaP, and 60 MPC BaP. In the control, the enzyme activity was 505.4 ± 54.4 and 423.2 ± 7.9 U/g (Figure 2f). The increase in GR activity was accompanied by a slight increase in the content of reduced glutathione in all pollution groups in the groups of 20 MPC BaP, 40 MPC BaP, and 60 MPC BaP, respectively. In the group of 20 MPC BaP, the increase in GR was 1.15 and 1.17 in the shoots and roots, respectively, while, as in the variants of the experiments of 40 and 60 MPC BaP, the increase in GR in the shoots was significant at 2.4 and 3.3 times, respectively. In the roots, in the experimental variants 40 and 60, the maximum concentration limit for BaP was 1.2 and 1.6 times, respectively.

The control values of GP were 45.7 ± 2.2 and 19.5 ± 0.5 (Figure 2g) U/g in shoots and roots, respectively. GP activity increased in the experimental variants 20 MPC BaP, 40 MPC BaP, and 60 MPC BaP by 3.8, 5.4, and 7 times in the shoots and in the roots by 3.2, 7.4, and 9.4 times, respectively.

The activity of glutathione-s-transferase (GST) in the control treatment was 170.2 ± 19 and 154 ± 27.5 U/g in the shoots and roots, respectively (Figure 2h). In the groups of 20 MPC BaP and 60 MPC BaP in the roots, a dose-dependent increase in GST by 3, 3.9, and 4.82 times was shown, respectively. When treated with 20, 40, and 60 MPCs of BaP, a similar increase in GST occurred in the aerial part by 2.4, 4.0, and 5.1 times, respectively.

### 2.5. Microscopic Examination

**Figure 5:**
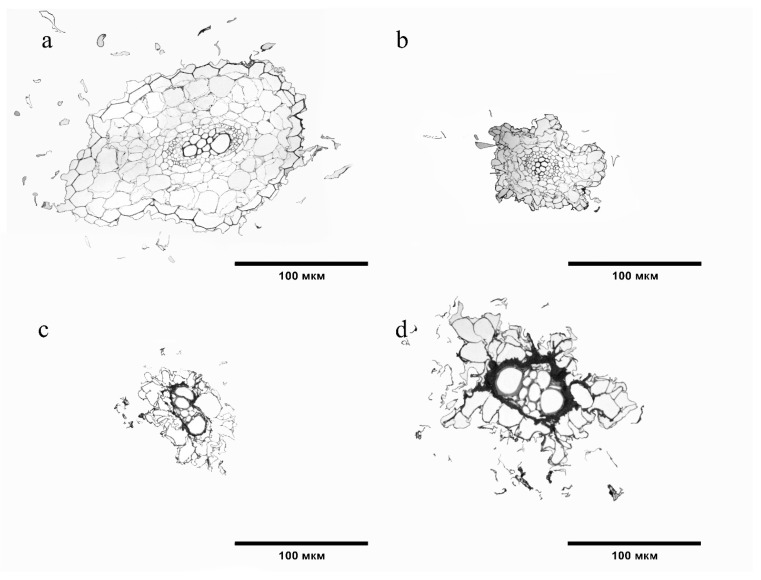
Cross sections of tomato roots: (**a**) Control; (**b**) 20 MPC BaP; (**c**) 40 MPC BaP; (**d**) 60 MPC BaP.

The structure of the *S. lycopersicum* root tissues in the variants with BaP differed relative to the control (Figure 5). In variant 20 MPC, the crustal layer of the *S. lycopersicum* root had a smaller area and size of its cells (Figure 5b). The greatest degree of change was observed at the highest doses of soil contamination (Figure 5c,d). A loose epiblema was observed, the walls of most cells were not corky, the area of the cortical layer was reduced, and the intercellular space and cell size of cortical parenchyma, endodermis, and xylem vessels were increased (Figure 5b,c).

**Figure 6:**
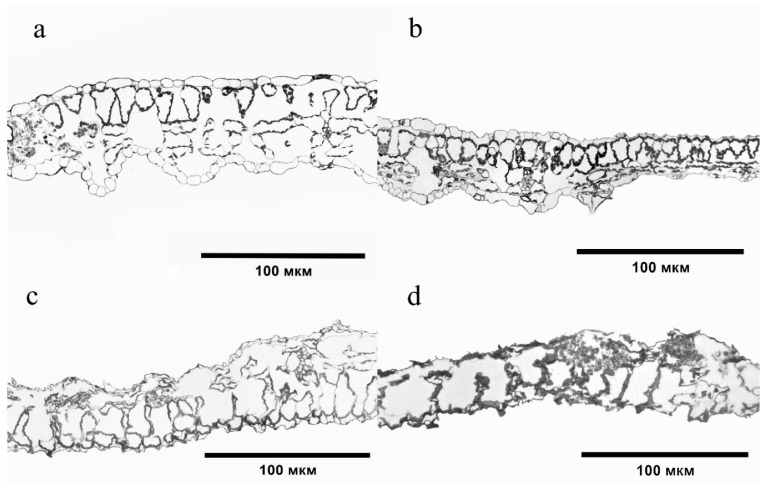
Cross sections of a *Solanum lycopersicum* leaf plate: (**a**) Control; (**b**) 20 MPC BaP; (**c**) 40 MPC BaP; (**d**) 60 MPC BaP.

Changes were also observed in the structure of tissues of the *S. lycopersicum* leaf plate, which were especially pronounced when exposed to 40–60 MPC of BaP. The epidermis became thinner, its turgor and cell size decreased, the intercellular space increased, and the structural and spatial organization of the chlorenchyma cells changed (Figure 6).

## 3. Discussion

The introduction of BaP into the soils led to its accumulation in *S. lycopersicum* at concentrations from 17.0 ng/g in the control variant of the experiment to 599 ng/g at a contamination of 60 MAC of BaP and had a dose-dependent effect. The ability to bioaccumulate BaP in plants occurred even at doses from 1 MPC of BaP [14] and its intensity was closely related to the pollutant concentration in the soil and the biophilicity of the BaP molecules. It was also shown that an increase in the concentration of BaP in the soil indirectly provoked a change in the morphobiometric parameters of *S. lycopersicum*. Therefore, in addition to the inhibition of root growth and plant height, a change in the shape of the leaf blade, shortening of the central vein, and twisting toward the adaxial side of the leaf, starting at 20 MPC BaP and progressing in each subsequent variant of the experiment, was noted. Changes in the generative organs of plants were observed in all variants of the experiment, however, in the variants of the experiment with 40 MPC of BaP, the vegetation stopped at the flowering stage, and at 60 MPC, the vegetation stopped in the active growth phase.

High levels of BaP accumulation provoke a toxic load in the cell wall and plant cell components, activating the processes of reactive oxygen species (ROS) overproduction. The high reactivity of ROS with proteins, lipids, carbohydrates, and nucleic acids causes not only a change in the morphobiological parameters, but also violated the redox homeostasis of plants. It has been found that the amount of chlorophyll pigments is also related to the physiology of stress. In response to an increase in ROS caused by the influence of BaP, a dose-dependent increase in the concentration of carotenoids occurred [15]. In the present study, the decrease in total chlorophyll did not have a clear pattern, but general trends were observed toward a decrease in the level of photosynthetic activity due to the accumulation of ROS products in the chloroplasts [16]. A distinctive feature of *S. lycopersicum* in the framework of the model study was a sharp decrease in the concentration of chlorophyll a in the experiment variant of 60 MPC of BaP, and 40 and 20 MPC of BaP each, which may indicate an excess of singlet oxygen or hydrogen peroxide. The removal of ROS in plants occurs not only through enzymatic, but non-enzymatic detoxification pathways, which include carotenoids [17]. The main function of carotenoids under BaP stress is to level the toxic effect of ^1^O_2_ on the photosynthetic apparatus of *S. lycopersicum.*

The accumulation of BaP caused an increase in the concentration of ROS by-products such as low molecular weight aldehyde—MDA. An increase in the amount of MDA can be used to judge the intensity of lipid peroxidation. The most pronounced biochemical is observed in the roots. BaP leads to damage to the cell membrane through the oxidation of the lipid structures of the membrane and the functional inactivation of various proteins. Due to an increase in the number of oxidized forms of various cell compounds, apoptosis occurs [18,19,20,21].

It was shown that due to the violation of the redox homeostasis of the cell, there was an increase in the concentration of O_2_^•–^, which was confirmed by histochemical staining of the leaves of *S. lycopersicum.* A significant increase in formazan blue spots was observed depending on the concentration of BaP in the plant tissues. An increase in the amount of ROS stimulated the overproduction of antioxidant enzymes, which was strongly associated with the presence of BaP. The balance of AS during the accumulation of BaP in the organs of *S. lycopersicum* shifted toward an increase in SOD activity in plants treated with different doses of BaP. The increase in PRX and APOX activity also correlated with the dose of the applied pollutant in all variants of the experiment [19]. A sharp increase in PRX and APOX may be associated with a sharp decrease in the activity level of CAT, which decomposes H_2_O_2_ to oxygen and water [22]. This may be due to the primary intense response of CAT to the pollutant in the germination and tillering phase, however, at later growth phases, the dominant enzymes that decompose hydrogen peroxide change [23]. Thus, the most pronounced response associated with H_2_O_2_ was recorded in the roots, which was confirmed by the accumulation of APOX in these parts of the plant, which is responsible for the buffer component of AS.

The results showed that the antioxidant response of APOX during the accumulation of BaP in plants is closely related to the activity of other enzymes of the AsA–GSH cycle, which plays an important role in the elimination of H_2_O_2_ under conditions of the excess growth of BaP. The total accumulation of APOX and PRX in *S. lycopersicum* explains the effective buffering of H_2_O_2_, reducing the toxic effect of ROS in all experimental variants [24].

The increases in GR, GST, and GP activity were the most significant compared to other enzymes, suggesting their dominant role in mitigating the BaP stress in *S. lycopersicum* [3,25]. This pattern of the accumulation of glutathione cycle enzymes in the aerial part of the plant indicates the activation of antioxidants in the aerial part of the plant [22,26], even though ROS products are mainly accumulated in the roots [27]. The amount of GSH increased in all variants of the experiment with soil contamination with BaP. It has been established that with an increase in the concentration of GSH, there is an increase in the activity of the enzymes of the glutathione cycle due to the acceptor function of GSH [28]. An increase in GST activity has a positive correlation with the accumulation of ROS and H_2_O_2_ in *S. lycopersicum* [28]. It has been established that with an increase in the GSH concentration, the GST activity also increases and has a positive correlation with the accumulation of ROS and H_2_O_2_ in *S. lycopersicum*. The data obtained correlated with the regularities revealed in *Z. mays* grown on oil-contaminated soil. The culture showed an increase in the number of transcripts encoding GST activity [29].

The greatest degree of change was observed at the highest dose of soil contamination. The epidermis became thinner, its turgor and cell size decreased, the intercellular space increased, and the structural and spatial organization of chlorenchyma cells changed (Figure 6). The reaction of plants to any environmental stress factor is species-specific. However, there are also similar changes in the structures of tissues of different species growing in PAH-contaminated soil. Thus, under the action of BaP in *Hordeum sativum*, degradation of the integumentary tissue of the roots and disturbance of the ordered arrangement of reduced cells of the primary cortex were observed [6]. Destruction of the epidermis and an increase in the size of the cortical cells with subsequent disruption of the originally concentric arrangement was found in *Pisum sativum* and *Z. mays* with the accumulation of polyarenes [30]. The same anatomical changes in the *S. lycopersicum* leaf structure were observed[31]. These structural changes may be the result of the disruption of transport processes or changes in the osmotic properties of cells induced by PAH toxicity [17].

It has been established that PAHs accumulate in the cell walls of root hairs at the first stages of contact with a pollutant and are then distributed inside the cells and organelles [31]. This is evidenced by changes at the cellular level. The reaction of plants to any environmental stress factor is species-specific. However, there are also similar changes in the tissue structures of different plant species growing in PAH-contaminated soil. Thus, under the action of BaP in *H. sativum*, degradation of the integumentary tissue of the roots and disturbance of the ordered arrangement of reduced cells of the primary cortex were observed [32]. Destruction of the epidermis and an increase in the size of the cortical cells with subsequent disruption of originally concentric arrangement was found in *Pisum sativum* and *Z. mays* with the accumulation of polyarenes [33]. The same anatomical changes in the tomato leaf structure were observed by Gratão [34]. These structural changes may be the result of the disruption of transport processes or changes in the osmotic properties of cells caused by PAH toxicity [33]. BaP significantly inhibited CAT activity, induced the ASC–GSH cycle, and some other markers of oxidative stress. Oxidative stress markers can reflect not only the toxic effect of BaP on the physiological and biochemical parameters of plants, but can also be used to prevent the toxic effect and possible remediation pathways at early stages.

## 4. Materials and Methods

### 4.1. Experimental Setup and Plant Growth Conditions

A model experiment was designed to reveal the toxic effect of BaP. The experiment was carried out in a climatic chamber while maintaining optimal lighting and humidity conditions. The soil for the model experiment was selected from a virgin area of the Persianovskaya Zapovednaya Steppe State Soil Sanctuary in the Rostov Region, Russia, located far from sources of anthropogenic load. In the model experiment, the top layer of 0–20 cm Haplic [35] was used on loess-like loams with the content of clay of 47.1%, dust of 26.8%, C_org_ of 3.7%, pH of 7.5, CaCO_3_ was 0.3%, and the cation exchange capacity was 37.1 mM (+)/100 g.

To implement a model experiment, the cleaned soil from the plant residues was sieved through a 2 mm diameter sieve, then placed in 5-L pots. BaP (99%, analytical grade, Sigma-Aldrich, Burlington, MA, United States) was introduced into the soil used in the model experiment. For the purity of the experiment, the pollutant was applied to the soil surface, simulating the natural intake. BaP was added in an acetonitrile solution. The soil was regularly moistened to a level of 60% of the total water capacity for better interaction of the soil with the BaP introduced into it and to create optimal growth conditions. The experimental scheme included a control variant with uncontaminated soil as well as the experimental variants artificially contaminated with BaP, in the amount of 400 ng/g—20 MPC, 800 ng/g—40 MPC, and 1200 ng/g—60 MPC [36]. The test culture was sown four months after the start of incubation.

A tomato (*Solanum lycopersicum* L.), an early-ripening variety “Bely naliv” 241 zoned in the south of Russia, was chosen as a test-culture. The number of plants in one pot was three plants. Sowing of seeds was carried out in vegetation pots. In the tillering phase, the plants were thinned out. Experiments were laid in 3-fold repetition. For the entire duration of the experiment, the temperature was maintained at +20–22 °C, natural light, and humidity in the soil, corresponding to the lowest field capacity. Sampling was carried out in the phase of full ripeness.

### 4.2. Measurement of Morphometric Parameters

Plants were taken from the soil and the nonradiometric parameters were measured in the phase of full ripeness: the length of the roots and leaves, plant and stem height, and productivity. For further studies, the plants were dried in air, the roots were removed from the aerial part, and ground with a mortar and pestle to a powdery consistency.

### 4.3. BaP Content in Plants Tissues

Determination of BaP in the plants was carried out by high performance liquid chromatography (HPLC). Sample preparation included the dissolution of the lipid fraction with a 2% solution of NaOH in alcohol, then the samples were extracted with hexane [37]. A round-bottom flask with the extract was placed in a rotary evaporator for further evaporation. After cooling, the precipitate formed during evaporation was dissolved in 1 mL of acetonitrile. Measurements were made on an Agilent 1260 liquid chromatograph with fluorometric detection (FL-3000, Santa Clara, USA), no later than three days after transferring the BaP concentrate into a vial (ISO 13877-2005 2005). The liquid phase was a mixture of acetonitrile (75%) and water (25%) with a flow rate of 0.5 mL/m. The BaP peak was recorded at λ = 409 nm.

### 4.4. Determination of Chlorophyll a,b, Total Chlorophyll, and Carotenoids

Freshly collected plant samples were taken to determine the chlorophyll a, b, total chlorophyll, and carotenoids. The sample was homogenized in 10 mL of 80% acetone. Samples were incubated at 4 °C in a dark place for 12 h. The filtrate was analyzed in a spectrophotometer at λ = 663 nm, λ = 645 nm, and λ = 470 nm (mg/g^−1^ dry weight (DW)). The calculation was carried out according to Equations (1) and (2) [37].

Chlorophyll a = (12.21 × A_663_) − (2.81 × A_645_) (1)

Chlorophyll b = (20.13 × A_645_) − (5.03 × A_663_) (2)

Total chlorophyll = 17.32 × A_645_ + 7.18 × A_663_ (3)

(4) $${\mathrm{Carotenoids}}_{\;(x\;+\;c)}=\frac{1000A_{470}-3.27\mathrm{ChlA}-104\mathrm{ChlB}}{229}$$

### 4.5. Localization of Superoxide in Situ

The determination of the intensity of superoxide production was carried out according to the Tewari method [38]. Leaves was placed with a solution of 25 mM 4-(2-hydroxyethyl)-1-piperazineethanesulfonic acid (HEPES, Merck, Darmstadt, Germany) buffer (pH 7.6) and 0.1 mg/mL-1 nitro blue tetrazolium (NBT, Thermo Fisher Scientific Inc., Waltham, MA, USA), incubated at 25 °C in a dark place for 2 h. Then, the sample was washed with 80% (v/v) ethanol heated to 70 °C for 10 min and placed in a solution containing lactic acid, phenol, and water (1:1:1).

### 4.6. Determination of Lipid Peroxidation

By measuring the content of malonic dialdehyde (MDA) in plant samples, the level of lipid peroxidation was determined. The definition is based on the reaction of MDA with thiobarbituric acid (TBA, Merck, Darmstadt, Germany) [39]. Measurements were made in a spectrophotometer at λ = 532 nm and λ = 600 nm, corrected for non-specific absorption [40]. The MDA content was expressed in μM/g DW and was calculated from its molar extinction coefficient of 155 mM^−1^cm^−1^.

### 4.7. Plant Sample Preparation to Determine the Activity of Antioxidant Enzymes and Total Protein

Samples of the roots and shoots in the amount of 100–500 mg were homogenized in liquid nitrogen. Homogenized plants were centrifuged at 12,000× *g* for 15 min at 4 °C. Using the obtained extract, the widespread activity of antioxidant enzymes was detected. The measurements were carried out on a spectrophotometer DU 800 (Beckman Coulter, Brea, CA, USA). All used reagents were purchased from Sigma-Aldrich, Burlington, MA, USA.

#### 4.7.1. Protein Definition

A sample of 0.1 mL of the extract obtained from the enzyme activity assays was placed in a test tube and 5 mL of Bradford’s [41] reagent was added, mixed, and left at room temperature for 20 min. Optical density was measured in a spectrophotometer λ = 595 nm. As a reference solution, instead of the sample, we used the same amount of buffer or extracting solution. The amount of protein was expressed in µg/mL.

#### 4.7.2. Determination of Proline (L: 210-189-3)

A total of 0.1 g of root tissue was homogenized with 4 mL dH_2_O, extracted at 100 °C for 10 min, and then the leaf volume was adjusted to 5 mL. The reaction mixture consisted of 750 µL of extract, 750 µL of acetic acid, and 750 µL of ninhydrin reagent. Next, incubation was carried out at 100 °C for 20 min [8,42]. The color intensity of the solution was measured with a spectrophotometer λ = 520 nm. The results of the analysis are expressed as U/mg of protein.

#### 4.7.3. Determination of Superoxide Dismutase Activity (EC 1.15.1.1)

Superoxide dismutase (SOD) was observed by the inhibition of the reduction reaction of nitro blue tetrazolium (NBT) during the oxidation of adrenaline to adenochrome at pH >7.0 under O2^•–^ production conditions [43]. The reaction mixture contained 1 mg polyvinylpyrrolidone (PVP), 6.11 mM HBT, 0.5 mM phenylmethylsulfonyl fluoride (PMSF), mM sodium phosphate buffer (pH 7.4), and 1 mM 1,4-dithiothreitol. The measurements were performed at a λ = 540 nm. Enzyme activity was expressed in U/mg protein × min.

#### 4.7.4. Determination of Catalase Activity (EC 1.11.1.6)

Catalase activity was determined [41] as the amount of H_2_O_2_ that did not decompose after incubation with catalase by registering the colored reaction product of the interaction of H_2_O_2_ with ammonium molybdate. The color intensity of the solution was measured with a spectrophotometer λ = 410 nm. The reaction mixture contained ammonium molybdate, 0.03% H_2_O_2_. Enzyme activity was expressed in nM/mg protein.

#### 4.7.5. Determination of Glutathione Reductase Activity (EC 1.8.1.7)

Glutathione reductase was determined by the reduction of oxidized glutathione with the participation of nicotinamide adenine dinucleotide phosphate (NADPH) [44]. The reaction mixture (pH = 8) contained tris(hydroxymethyl) aminomethane (Tris), Na-EDTA, supernatant, and 0.4% NADPH solution. The color intensity of the solution was measured with a spectrophotometer at λ = 340 nm. The outcome was expressed in U/g protein.

#### 4.7.6. Determination of Glutathione Peroxidase Activity (EC 1.11.1.9)

The activity of glutathione peroxidase was assessed by the content of GSH in the samples before and after incubation with the substrate during the color reaction with 5,5’-dithiobis-2-nitrobenzoic acid (DTNBA) [45]. The reaction mixture consisted of 0.73 mL 0.1 M Tris (pH 8.5), Na-EDTA, 100 mM PMSF, 0.2 mL extract, 0.07 mL HTB, and 0.2 mL 20% TCA. Enzyme activity was measured in U/g protein.

#### 4.7.7. Determination of Ascorbate Peroxidase Activity (EC 1.11.1.11)

Ascorbate peroxidase (APOX) was noted by the decomposition reaction of H_2_O_2_ APOX with the reaction products in the form of H_2_O and dehydroascorbate [46] in the studied samples. The reaction mixture contained K, Na-phosphate buffer (pH 7.0), 17 mM ascorbic acid, 100 mM PMSF, PVP, extract, and 0.06% H_2_O_2_. The measurements were carried out at λ = 290 nm for 3 min. The results were expressed mM/mg protein.

#### 4.7.8. Determination of Total Peroxidase Activity (EC 1.11.1)

The peroxidase activity (PRX) of extracellular peroxidases was determined calorimetrically [46]. The reaction mixture contained 0.2 M Na-acetate buffer, 0.01% benzidine hydrochloride, supernatant, and 0.3% H_2_O_2_. The color intensity of the solution was measured at λ = 600 nm. PRX was expressed in U/mg protein.

#### 4.7.9. Glutathione-S-Transferase (EC2.5.1.18)

The activity of glutathione-s-transferase was analyzed by the reaction of reduced glutathione (GSH) and 1-chloro-2,4-dinitrobenzene (CDNB) with the formation of glutathione-S-conjugates [30]. The reaction mixture contained 0.1 K-phosphate buffer (pH = 6.5), 0.015 M GSH and the supernatant. The reaction was initiated with 0.015 M 1-chloro-2,4-dinitrobezol. Detection was performed at λ = 340 nm. Enzyme activity was measured in U/g protein.

#### 4.7.10. Definition of Glutathione

The content of total glutathione in the samples was estimated during the color reaction during the formation of a complex of 5,5′-dithiobis-2-nitrobenzoic acid (DTNBA) and GSH. To evaluate the content of GSSG, 2-vinylpyridine was used, which binds to GSH [46]. The reaction mixture consisted of 0.18% Na-EDTA in 0.5 M sodium phosphate buffer, 0.16% NADPH, and 0.12% 5,5-dithiobis (2-nitrobenzoic acid), extract, and glutathione reductase. The results of changing the optical density of the solution were measured spectrophotometrically at λ = 412 nm. Enzyme activity was measured in μ/mg protein.

### 4.8. Light Microscopy

Histopathological evaluation was performed on tissue from the central part of the leaf (2 × 2 mm) and in the zone of root hairs (2 mm). The preparation of the histological specimens consisted of the following stages: plant tissue sampling, fixing, dehydration, embedding with polymerization of the prepared blocks, sectioning, and staining with toluidine blue [6]. Evaluation of the histological slides was performed using a Mikmed-6 microscope.

### 4.9. Statistical Analysis

Microsoft Excel 2016 and SPSS-13 (IBM SPSS Statistics 23) programs were used for data processing and statistical analysis. The coefficient of variation was <10%. Reliability of differences between the values of the frequency of occurrence was *p* < 0.05. The whiskers on the columns in the figures indicate the standard deviation level.

## 5. Conclusions

The toxic effect of BaP manifested itself from 20 MPC, and increased with the dose of the applied pollutant. The results demonstrate the effect of BaP on all morphophysiological, structural, and biochemical parameters of the test culture. A decrease in photosynthetic activity and a violation of the redox homeostasis of *S. lycopersicum* associated with oxidative stress were diagnosed: accumulation of ROS and their by-products, and the overproduction of antioxidant enzymes in all variants of the experiment. Despite a number of general trends in the response to stress caused by BaP, a significant difference was found in the nature of oxidative stress and a change in the dominant pathways of H_2_O_2_ detoxification from CAT to ASC-GSH and PRX. Thus, BaP toxicity is based on enzymatic toxicity, membranotropic action, and oxidative stress. Further studies will provide the most complete information regarding the most relevant markers of oxidative stress for PAHs, monitoring the impact of BaP in the long-term.

## Data Availability

Not applicable.

## Abbreviations

BaP	Benzo[a]pyrene
MPC	Maximum permissible concentration
CAT	Catalase
SOD	Superoxide dismutase
MDA	Malonic dialdehyde
PRX	Peroxidase
APOX	Ascorbate peroxidase
GP	Glutathione peroxidase
PAHs	Polycyclic aromatic hydrocarbons
GSH	Gluthatione
AS	Antioxidant system
HPLC	High performance liquid chromatography
HEPES	4-(2-Hydroxyethyl)-1-piperazineethanesulfonic acid
NBT	Nitro blue tetrazolium
TBA	Thiobarbituric acid
DW	Dry weight
PVP	Polyvinylpyrrolidone
PMSF	Phenylmethylsulfonyl fluoride
NADPH	Nicotinamide adenine dinucleotide phosphate
Tris	Tris (hydroxymethyl) aminomethane
CDNB	1-Chloro-2,4-dinitrobenzene
GST	Glutathione-S-transferase
